# Case Report: A Promising Treatment Strategy for Noninfectious Uveitis

**DOI:** 10.3389/fphar.2021.784860

**Published:** 2022-01-18

**Authors:** Xiao-Bao Liu, Lu-Shi Tang, Jing-Wen Chen, Chang-Song Lin, Qiu-Hong Liu, Qiang Xu

**Affiliations:** ^1^ The First Clinical Medical College, Guangzhou University of Chinese Medicine, Guangzhou, China; ^2^ Department of Rheumatology, The First Affiliated Hospital of Guangzhou University of Chinese Medicine, Guangzhou, China; ^3^ Department of Ophthalmology, The First Affiliated Hospital of Guangzhou University of Chinese Medicine, Guangzhou, China

**Keywords:** noninfectious uveitis, tofacitinib, case report, JAK inhibitor, glucocorticoid drugs

## Abstract

**Background:** Uveitis refers to inflammation in the uvea, retina, retinal blood vessels, and vitreous, which can lead to irreversible eye damage and permanent vision loss. Glucocorticoid drugs are the first-line treatment, but side effects, such as obesity and hyperglycemia, can occur. Therefore, biologics have become a new treatment choice.

**Case Presentation:** A 18-year-old girl developed eye pain and was diagnosed with binocular uveitis. Prednisone 50 mg was administered once a day, and the redness and pain in both eyes improved. Later, the prednisone dose was gradually reduced, and treatment was discontinued 3 years ago. Two years ago, the patient’s condition relapsed, with both eyes becoming red and painful. She was administered prednisone 20 mg once daily and adalimumab. Visual acuity in both eyes continued to progressively decrease, accompanied by cataracts. At the same time, the patient experienced complications, including obesity and hyperglycemia. Subsequently, a new treatment regimen, oral prednisone 20 mg once a day, tofacitinib 5 mg twice a day, and methotrexate 10 mg once a week, as well as the use of insulin to control blood sugar, was initiated. One month later, the patient’s redness and eye pain eased, and her vision gradually improved. The dosage of prednisone was gradually reduced to 5 mg once daily. At the same time, her blood sugar returned to normal, and insulin was stopped.

**Outcomes:** The patient was treated with tofacitinib for 10 months. Subsequently, her best-corrected visual acuity of the right eye rose from 0.06 to 0.075, and the best-corrected visual acuity of the left eye rose from CF/30 cm to CF/100 cm. Redness and eye pain were relieved, her glucocorticoid consumption reduced from 15 to 2.5 mg, and her blood sugar gradually normalized.

**Conclusion:** This case study shows that tofacitinib relieves ocular inflammation in patients with uveitis and improves eyesight. We believe that JAK inhibitors could be another treatment option for noninfectious uveitis in patients who do not respond to conventional anti-TNF-α inhibitors (such as adalimumab).

## Introduction

Uveitis refers to inflammation that occurs in the uvea, retina, retinal blood vessels, and vitreous, which can lead to irreversible eye damage and permanent vision loss (De Smet et al., 2011). Uveitis can be sub classified as anterior uveitis, intermediate uveitis, posterior uveitis, and pan uveitis, according to the anatomical location of the inflammation. The etiology of uveitis can be divided into infectious and noninfectious uveitis (Zierhut et al., 2007). The incidence of noninfectious uveitis was ranked first. In a cross-sectional study in Austria, a total of 2,619 children and adult patients with uveitis were included; the incidence of anterior uveitis was 59.9% and the incidence of noninfectious uveitis was 81% (Barisani-Asenbauer et al., 2012).

Noninfectious uveitis is an autoimmune inflammatory disease mediated by T cells. Melanin-related antigens are rich in the uvea which contains slow choroidal blood, making it susceptible to autoimmune attacks. Antigens in normal eye tissues, such as retinal S antigen and melanin-related antigen, can be recognized by the immune system when the immune function is disordered, and induce an immune response mediated by Th17 cells, Th1 cells, and cytokines produced by immune cells. This immune response results in the localized inflammation called uveitis (Yoshimura et al., 2007; De Smet et al., 2011; Amadi-Obi et al., 2017).

Cytokines play a central role in the pathogenesis of inflammation and autoimmune diseases. Many of these cytokines send signals via the tyrosine kinase signal transducer and activator of transcription (JAK-STAT) pathway. The non-receptor tyrosine kinase (JAK) family is a key intracellular component of cytokine signaling (Virtanen et al., 2019). The JAK kinase family includes four members: JAK1, JAK2, JAK3, and TYK2. Tofacitinib is a JAK1/JAK3 inhibitor approved by the U.S. Food and Drug Administration (FDA) in November 2012 for the treatment of rheumatoid arthritis (Yoshimura et al., 2007). Related animal studies have shown that tofacitinib can inhibit the development of autoimmune uveitis and reduce the proportion of Th1 cells (Bing et al., 2020).

In this study, we report the case of a patient with refractory noninfectious anterior uveitis who underwent treatment with tofacitinib, which we think it could be the first reported case that noninfectious uveitis treated with JAK inhibitor. We had an in-depth discussion with the patient. Informed consent was signed by the patient for the treatment strategy and reporting the case.

## Case Presentation

An 18-year-old girl developed eye pain 5 years ago without obvious cause, and her rheumatism and immune-related indicators at Guangzhou Zhongshan Eye Center were all negative. She was thus diagnosed with binocular uveitis and was administered prednisolone 50 mg once a day. After the treatment, the redness and pain in both eyes improved, and the prednisone dose was gradually reduced until treatment was stopped 3 years ago. However, 2 years ago, the patient’s condition relapsed, and red eye pain again occurred. She was administered prednisone 20 mg once a day and adalimumab 40 mg every 2 weeks; unfortunately, the visual acuity in both eyes continued to progressively decrease, and was accompanied by cataracts. The cataract grade of her right eye was N1C2P1 according to the classification standard of LOCSII lens opacity, and was grade I according to the Emery-Little hardness scale. She underwent cataract phacoemulsification and intraocular lens in the left eye; however, postoperative inflammation recurred, accompanied by pupil closure. Prednisone 20 mg was administered once a day, and methotrexate, mycophenolate mofetil, and cyclosporine were administered successively, but her condition remained uncontrolled. Her visual acuity gradually declined, and complications such as obesity (BMI 39.1) and hyperglycemia were observed. Her weight increased from 42.5 kg 5 years ago to 104 kg, and her fasting blood sugar level reached as high as 16 mmol/L .

**FIGURE 1:**
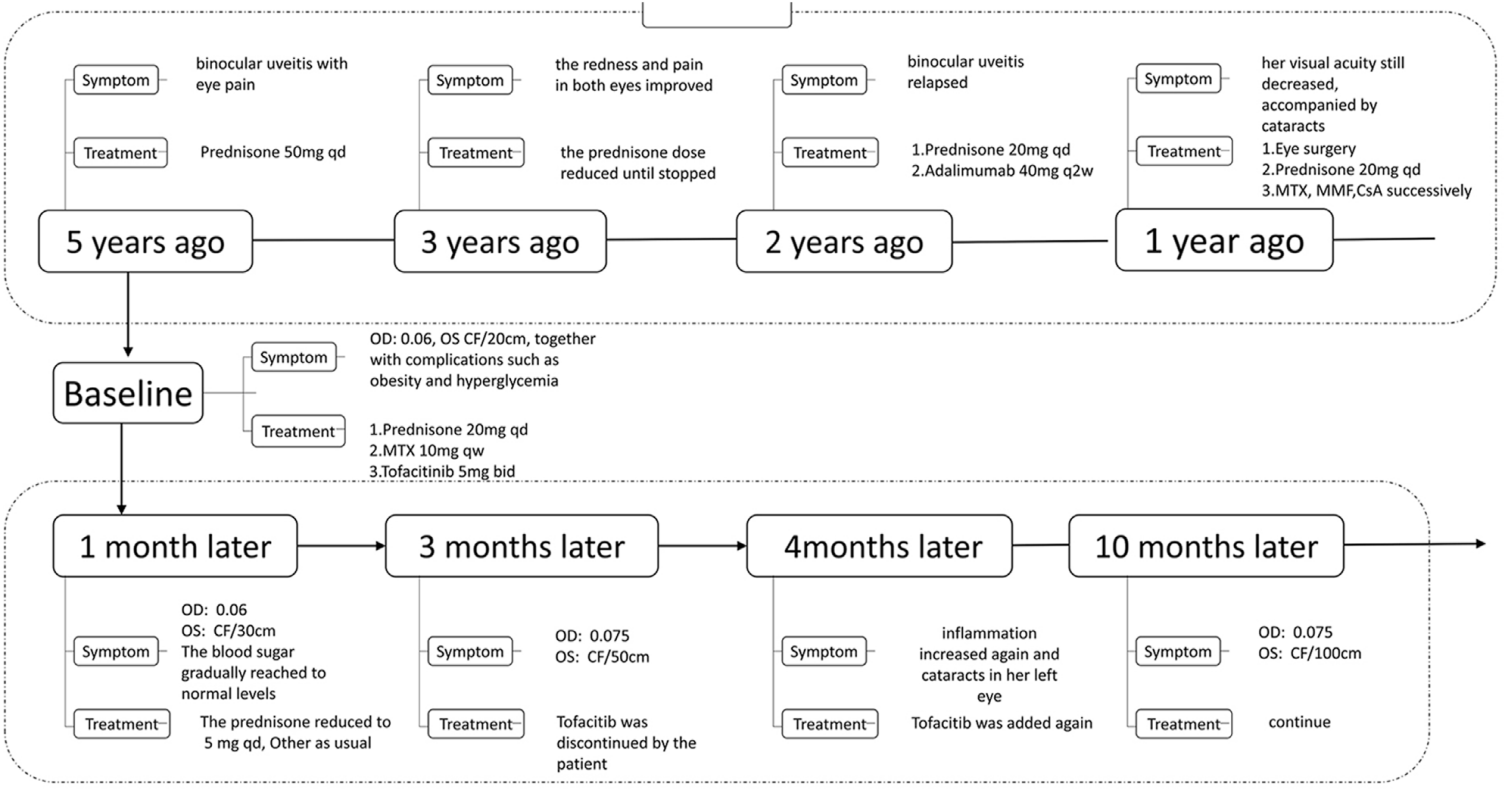
Treatment progress.

For further treatment, the patient came to the Department of Rheumatology, the First Affiliated Hospital of Guangzhou University of Traditional Chinese Medicine 8 months ago, and was hospitalized. For diagnosis, she was perfected with visual acuity, intraocular pressure examination, anterior segment and fundus examination under slit lamp, B-ultrasound examination and immunological examination. Tests revealed she was HLA-B27 (-), ANA (-), DsDNA (-), anti-ENA antibody profile negative, and her best corrected visual acuity (BCVA) was OD: 0.06, OS CF/20 cm, under slit lamp microscopy. The patient’s right conjunctiva was mildly hyperemic, the cornea was transparent, with retrocorneal deposits (+), anterior chamber empyema, Tyndall phenomenon (-), irregular pupils, adhesions behind the iris, and opacity of the lens. Mild conjunctival hyperemia in the left eye, transparent cornea, retrocorneal deposits (+), anterior chamber empyema, Tyndall phenomenon (-), pupil membrane closure, pupil light reflection disappeared, posterior iris adhesion, and intraocular lens reign. Considering that she had no clear history of infection, she was diagnosed as noninfectious chronic anterior uveitis (Figure 1).

**FIGURE 2:**
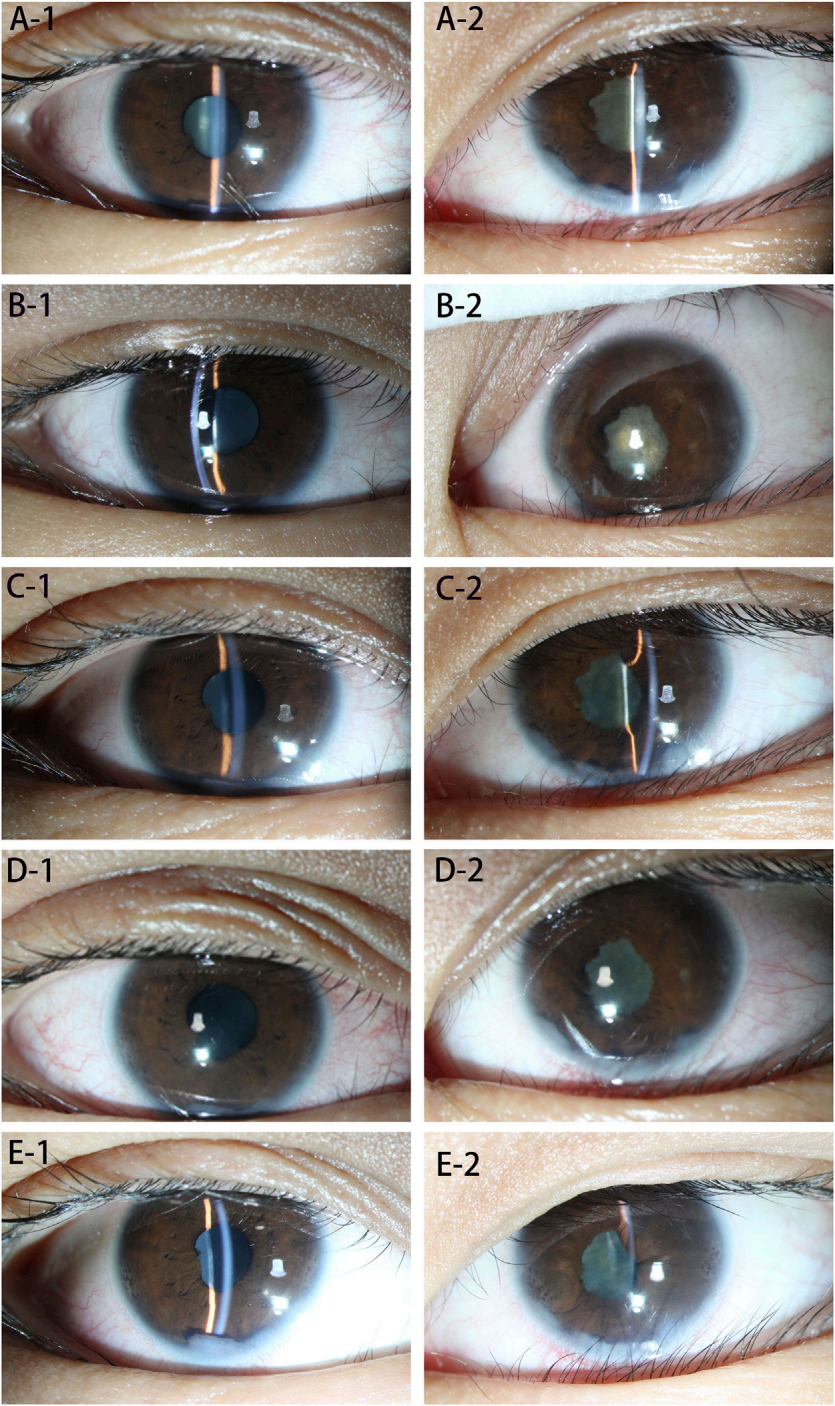
**(A–E)**: Eye examination at each stage. **(A-1)** Shows the right eye before treatment, showing mild mixed conjunctival hyperemia, anterior chamber empyema, posterior corneal deposits (+), posterior iris adhesions, and mild lens opacity. **(A-2)** Shows the left eye before treatment, showing mild mixed conjunctival hyperemia, anterior chamber empyema, posterior corneal deposits (+), posterior iris adhesion and pupil closure. **(B-1)** Shows the right eye 1 month after the treatment. Conjunctival hyperemia, anterior chamber empyema, posterior corneal deposits (+), and posterior iris adhesions did not change significantly, and the degree of lens opacity was less than before. **(B-2)** Shows the left eye 1 month after treatment; there was no obvious change compared with the previous picture. **(C-1)** Shows the right eye 2 months after treatment; there was no significant change compared with the previous picture. **(C-2)** Shows the left eye 2 months after treatment, with mild mixed conjunctival hyperemia, anterior chamber empyema, posterior corneal deposits (+), posterior iris adhesions compared to before, no obvious changes, and fiber exudation in the pupil area. The film was absorbed and became lighter than before. **(D-1)** Shows the right eye, 3 months after treatment. The patient’s conjunctival hyperemia was worse than before, and the rest had no obvious changes. **(D-2)** Shows the left eye 3 months after treatment, the conjunctival hyperemia was worse than before, and the rest showed no obvious changes. **(E-1)** Shows the right eye, 10 months after treatment. Conjunctival mixed congestion is lessened, anterior chamber empyema, posterior corneal deposits, and the front image showed no obvious changes; the posterior iris adhesions were loosened compared with the front, the lens surface was pigmented, and the lens was slightly opaque. **(E-2)** Shows the left eye, 10 months after treatment. Conjunctival mixed conjunctival hyperemia was lessened, posterior iris adhesions were aggravated, and the rest had no obvious changes.

The new treatments were given to her: oral prednisone 20 mg once a day, tofacitinib 5 mg twice a day, and methotrexate 10 mg once a week, together with pranoprofen eye drops (1 drop, three times daily) topically to control inflammation on the basis of medication at the beginning. At the same time, insulin was used to control her blood sugar. One month later, the patient’s redness and pain in both eyes were relieved, and the best-corrected visual acuity of the left eye rose from 20 to 30 cm. Therefore, the dosage of prednisone was gradually reduced to 5 mg once a day, and her blood sugar returned to normal, and insulin was stopped. Her weight dropped to 101 kg, and after 3 months, the patient felt better and stopped using tofacitinib by herself for economic reasons, which led to an increase in inflammation and cataracts in her left eye. At that time, compound tropicamide eye drops (1 drop, four times daily) was used to control the inflammation. After tofacitinib was added again, she felt better, and her eyesight improved and the eye drops were gradually stopped. After treatment for a total of 10 months, her best-corrected visual acuity of the right eye was 0.075, and the best-corrected visual acuity of the left eye rose to 100 cm. Otherwise, her blood sugar and weight returned to normal levels (Figure 2).

The changes in her eyes over the past 10 months of treatment are shown in Table 1.

**TABLE 1:** Best corrected vision and intraocular pressure of each stage.

Time	Best corrected vision	Intraocular pressure (mmHg)
Baseline	OD: 0.06	OD: 8
	OS: CF/20 cm	OS: 7
1 month later	OD: 0.06	OD: 12
	OS: CF/30 cm	OS: 11
2 months later	OD: 0.075	OD: 12
	OS: CF/50 cm	OS: 12
3 months later	OD: 0.075	OD: 13
	OS: CF/50 cm	OS: 11
10 months later	OD: 0.075	OD: 12
	OS: CF/100 cm	OS: 10

## DISCUSSION

Glucocorticoid drugs are still the first-line treatment for noninfectious uveitis (Pavesio and Decory, 2008). Although glucocorticoids are effective in the treatment of such autoimmune-related diseases, their side effects are also a concern. Data show that more than 90% of patients have steroid-related complications for more than 60 days (Curtis et al., 2006). Long-term use of glucocorticoids will not only cause systemic side effects such as endocrine, neuropsychiatric, gastrointestinal, musculoskeletal, cardiovascular, and skin, but also ocular complications (Oray et al., 2016). Cataract (11–15%) and glaucoma (12.8%) are the two most common ophthalmic side effects of glucocorticoids (Fel et al., 2012). In this case, complications such as glucocorticoid-related cataracts, obesity, and hyperglycemia occurred during the course of glucocorticoid administration to control ocular inflammation.

When conventional immunosuppressive drugs are ineffective or have developed tolerance, biologics are the best choice for treatment, of which anti-TNF-α inhibitors are the most widely used (Leclercq et al., 2020). Adalimumab has been approved by the FDA to treat patients with steroid-dependent or noninfectious uveitis with steroid contraindications (Dick et al., 2018). Infliximab is recommended as the first-line treatment for Behçet’s disease-related uveitis (Levy-Clarke et al., 2014). However, the failure rate of treatment with anti-TNF-α inhibitor drugs remains high, at approximately 30% (Leclercq et al., 2020). Llorenc et al. analyzed 392 noninfectious uveitis patients treated with adalimumab. They found that the drug retention rate of adalimumab was 92.7% at 6 months, 87.68% at 12 months, 76.31% at 24 months, and 54.28% at 60 months (Llorenç et al., 2020). In this patient, adalimumab was injected subcutaneously (40 mg, every 2 weeks) for over half a year, but this treatment failed to control the progression of uveitis, and the patient’s vision continued to decline.

Non-receptor tyrosine kinase (JAK) is a key intracellular component of the cytokine signaling pathway. The JAK-STAT pathway is the main intracellular pathway regulating the cytokine I and II receptors. This signaling pathway is composed of four non-receptor tyrosine kinases (JAK1,2,3 and TYK2) and seven signal transducers and transcription activation (STAT1, STAT2, STAT3, STAT4, STAT5a, STAT5b, and STAT6). After the cytokine binds to its receptor, the receptor subunits are rearranged, which further activates JAK. Activated JAK phosphorylates this receptor, and the phosphorylated receptor binds to STAT, resulting in the activation of STAT and the transfer of activated STAT to the nucleus, where it binds to promoter sequences to regulate target gene expression (Virtanen et al., 2019).^1^

Tofacitinib is a first-generation JAK inhibitor that inhibits the enzymatic activity of JAK1 and JAK3, thereby blocking their dependent cytokine signaling pathways (Gadina, 2014). Currently, tofacitinib has been approved by the FDA for the treatment of rheumatoid arthritis (Virtanen et al., 2019) and psoriatic arthritis (Gladman et al., 2017). In addition, many studies are ongoing to explore the potential efficacy of tofacitinib in the treatment of uveitis.

Jin Bing et al. conducted an animal study using tofacitinib to treat mice with experimental autoimmune uveitis. They found that tofacitinib delayed the development of experimental autoimmune uveitis by inhibiting pathogenic Th1 cells, reducing the secretion level of interferon-γ, and strengthening the development of non-pathogenic Th17 cells (Bing et al., 2020). Miserocchi et al. reported four cases of JAK inhibitors used in the treatment of juvenile idiopathic arthritis (JIA) with uveitis. Three patients were treated with baricitinib, and one case was treated with tofacitinib. The study found that all the ocular inflammation associated with uveitis was controlled (Miserocchi et al., 2020).

This case study showed that tofacitinib can significantly alleviate ocular inflammation in some patients with uveitis, resulting in improvements in vision. We observed no obvious adverse effects during the follow-up period; however, this is only a case study. Actually, we also can not 100% confirm that the efficacy was from the JAK inhibitor monotherapy or JAK inhibitor with methotrexate. More clinical trials are needed to verify the efficacy and safety of tofacitinib in the treatment of noninfectious uveitis. In general, JAK inhibitors are expected to become a new treatment option for patients with noninfectious uveitis, especially for patients in whom other types of biological agents are ineffective and fail to control disease.

## Conclusion

This case study shows that tofacitinib can relieve ocular inflammation in patients with uveitis, especially to those who do not respond to conventional anti-TNF-α inhibitors (such as adalimumab), resulting improvements in eyesight. We believe that JAK inhibitors could be another treatment option for noninfectious uveitis patients who do not respond to conventional anti-TNF-α inhibitors (such as adalimumab).

## Data Availability

The original contributions presented in the study are included in the article/Supplementary Material, further inquiries can be directed to the corresponding authors.^1^

## Footnotes

1
https://www.jianguoyun.com/p/DR6nLVwQgOTwCRiUnpAE
